# Dietary patterns and risk for gastric cancer: A case-control study in residents of the Huaihe River Basin, China

**DOI:** 10.3389/fnut.2023.1118113

**Published:** 2023-01-23

**Authors:** Xiaomin Wu, Qian Zhang, Hong Guo, Ning Wang, Xueqi Fan, Bin Zhang, Wei Zhang, Wanying Wang, Zhongze Fang, Jing Wu

**Affiliations:** ^1^Department of Epidemiology and Biostatistics, School of Public Health, Tianjin Medical University, Tianjin, China; ^2^National Center for Chronic and Non-communicable Disease Control and Prevention, Chinese Center for Disease Control and Prevention, Beijing, China; ^3^Department of Toxicology and Sanitary Chemistry, School of Public Health, Tianjin Medical University, Tianjin, China; ^4^School of Public Health, Inner Mongolia Medical University, Hohhot, China; ^5^Key Laboratory of Liaoning Tumor Clinical Metabolomics, Jinzhou, Liaoning, China

**Keywords:** dietary pattern, gastric cancer, factor analysis, unconditional logistic regression, case-control study

## Abstract

**Aim:**

Evidence linking dietary patterns and the risk of gastric cancer was limited, especially in Chinese populations. This study aimed to explore the association between dietary patterns and the risk of gastric cancer in residents of the Huaihe River Basin, China.

**Methods:**

The association between dietary patterns and the risk of gastric cancer was investigated through a case-control study. Dietary patterns were identified with factor analysis based on responses to a food frequency questionnaire (FFQ). Gastric cancer was diagnosed according to the International Classification of Diseases, 10th Revision (ICD 10). Odds ratios (*OR*s) and 95% confidence intervals (*CI*s) were calculated across the tertiles of dietary pattern scores using unconditional logistic regression models.

**Results:**

A total of 2,468 participants were included in this study. Six main dietary patterns were extracted, and those patterns explained 57.09% of the total variation in food intake. After adjusting for demographic characteristics, lifestyle factors, individual disease history, family history of cancer and *Helicobacter. Pylori* (*H. pylori*) infection, comparing the highest with the lowest tertiles of dietary pattern scores, the multivariable *OR*s (95% *CI*s) were 0.786 (0.488, 1.265; *P*_trend_ < 0.001) for the flavors, garlic and protein pattern, 2.133 (1.299, 3.502; *P*_trend_ < 0.001) for the fast food pattern, 1.050 (0.682, 1.617; *P*_trend_ < 0.001) for the vegetable and fruit pattern, 0.919 (0.659, 1.282; *P*_trend_ < 0.001) for the pickled food, processed meat products and soy products pattern, 1.149 (0.804, 1.642; *P*_trend_ < 0.001) for the non-staple food pattern and 0.690 (0.481, 0.989; *P*_trend_ < 0.001) for the coffee and dairy pattern.

**Conclusions:**

The specific dietary patterns were associated with the risk of gastric cancer. This study has implications for the prevention of gastric cancer.

## Introduction

As the fifth most frequently diagnosed cancer and the third leading cause of cancer deaths worldwide, gastric cancer has sparked concern (1). Although gastric cancer rates were significantly declining worldwide, the pace has been variable in different regions (1). China has the largest number of incident gastric cancer cases in 2020 (2). The disease burden remained heavy in China (3–5). The incident gastric cases in China accounted for 48.26% of the global burden in 2019 (4).

Infectious factors, environmental risk factors and lifestyle were all linked with an increased risk of gastric cancer (1). Specifically, dietary factors were associated with an increased risk of gastric cancer, such as specific food consumption (such as meat, alcohol and so on), a high-salt diet, a low-vitamin A and C diet and consuming large amounts of smoked or cured food (6–9).

Food factors of diet are correlated with each other, making them difficult to appear in isolation (10). The dietary pattern could provide a more comprehensive assessment of the collective importance of different dietary factors (10–12). The dietary pattern has been applied to summarize and capture the overall dietary exposure and variations (13). The results of the association between dietary patterns and gastric cancer were mixed (10, 14–17). Given that, the association between dietary patterns and the risk of gastric cancer was examined in residents of the Huaihe River Basin, China, in the present study. The focus should be on modifiable factors as early as possible, which could show high effectiveness in preventing gastric cancer at a low cost.

## Materials and methods

### Study population

The field investigation was performed in 14 counties or districts of the Huaihe River Basin in 2021. All participants were chosen from local residents. We used data from the local cancer registry to select gastric cancer cases. Controls were randomly selected from local residents. The study complied with the principles of the Declaration of Helsinki and was approved by the Ethics Committee of National Center for Chronic and Noncommunicable Disease Control and Prevention, Chinese Center for Disease Control and Prevention. In addition, all participants were aware of the research purpose and provided written informed consent.

### Inclusion criteria and exclusion criteria

Cases were included according to the following criteria: (1) cases were diagnosed with gastric cancer from January 1, 2020, to July 31, 2021; (2) the absence of mental disorders or cognition dysfunction; (3) Subjects who have lived in the local area for more than 10 years; (4) Subjects were not to be given a diagnosis of other malignant tumors; (5) The diagnostic criteria were consistent with the 2019 Chinese Cancer Registry Annual Report (ICD-10/C16). Cases were excluded according to the following criteria: (1) cases were diagnosed as gastric cancer before January 1, 2020; (2) Subjects who were unable to participate in the investigation due to health problems; (3) Subjects who were unable to cooperate in the investigation due to serious illness or other reasons; (4) having a medical disease that influenced language function or movement; (5) Subjects who have not lived in the local area for more than 10 years, or could not return to the local area for other reasons such as migrant work; (6) Subjects were to be given a diagnosis of other types of cancers; (7) The diagnostic criteria were different from the 2019 Chinese Cancer Registry Annual Report.

Controls were included according to the following criteria: (1) The age difference between cases and controls was within 5 years; (2) the absence of mental disorders or cognition dysfunction; (3) Subjects who have lived in the local area for more than 10 years; (4) Subjects were not to be given a diagnosis of malignant tumors. Controls were excluded according to the following criteria: (1) The age difference between cases and controls was over 5 years; (2) Subjects who were unable to participate in the investigation due to health problems; (3) Subjects who were unable to cooperate in the investigation due to serious illness or other reasons; (4) having a medical disease that influenced language function or movement; (5) Subjects who have not lived in the local area for more than 10 years, or could not return to the local area for other reasons such as migrant work; (6) Subjects were to be given a diagnosis of malignant tumors.

### Data collection

Information on demography, lifestyle factors and medical history was collected by standard questionnaires. Dietary intake was assessed using a food frequency questionnaire (FFQ) including 16 food groups. The FFQ recorded about the specific mean consumption frequency (times/day, times/week, times/month, and times/year) and the specific mean consumption amount of selected food items during the previous months. Education was categorized into middle school or less, high school and college or more. Occupation was categorized into four categories: professional and administrative, office, sales and service, laborer and agriculture, unemployed, and others. Smoking status was categorized into never smoker, former smoker and current smoker (every day and sometimes). Drinking status was categorized into drinker and non-drinker. Health conditions including medical history, family history of cancers and so on were examined by clinicians. Height and weight were measured and recorded by a trained investigator. Body mass index (BMI) was calculated as weight in kilograms divided by height in meters squared (kg/m^2^).

### Assessment of dietary patterns

Factor analysis (principal components analysis) was used to derive major dietary patterns and to determine factor loadings for each food subgroups (in g/d). Evaluation of the eigenvalues and scree plot test was used to determine the number of retained factors. Varimax rotation was used to maintain uncorrelated factors and enhance interpretability. Six factors (eigenvalues >1.0) could describe distinctive dietary patterns of the study population. Food groups with absolute factor loadings >0.4 were the main contributors to the dietary patterns. Factors were named descriptively according to the food groups showing high loading (absolute value) with respect to each dietary pattern as follows: flavor, garlic and protein pattern (factor 1), fast-food pattern (factor 2), vegetable and fruit pattern (factor 3), pickled food, processed meat products and soy products pattern (factor 4), non-staple food pattern (factor 5), and coffee and dairy pattern (factor 6, Table 2), which totally explained 57.09% of the variance in food intake. For each participant, the factor scores for each dietary pattern were calculated by summing the consumption of the food groups weighted by their factor loadings and a higher score indicated stricter adherence to that dietary pattern.

### Assessment of *Helicobacter. pylori* (*H. pylori*) infection

All participants completed the *H. pylori* questionnaire. It consisted of four main modules: (1) knowledge of *H. pylori*; (2) infection conditions of *H. pylori*; (3) treatment of *H. pylori*; (4) infection conditions of *H. pylori* in the family. All questionnaires were completed by specialized medical personnel to ensure their reliability, authenticity, and completeness. Then, all participants received the *H. pylori* test. *H. pylori* infection was detected by the C13 urea breath testing (UBT), which was administered by trained investigators following the instructions. If subjects were not aware own infection condition, the detection result was used to indicate their *H. pylori* infection. Additionally, participants were categorized as positive or negative.

### Statistical analysis

SAS version 9.4 for windows (SAS, Inc.) was used to analyze all data. All continuous variables were described by mean and standard deviation, whilst categorical variables were described by number of cases and percentage. The general characteristics between the case and control subjects were compared by using Student's *t*-test for continuous variables and the chi-square test for categorical variables. The factor scores for each dietary pattern were categorized into tertiles. Odds ratios (*OR*s) and 95% confidence intervals (*CI*s) were calculated across the tertiles of the dietary pattern scores using the unconditional logistic regression model. The lowest tertile of each pattern score was used as the reference. Five models were developed. Model 1 was a crude model. Model 2 was adjusted for age (continuous: y), gender (male or female), and BMI (continuous: kg/m^2^). Model 3 was additionally adjusted for education lever (middle school or less, high school, college, or more), occupation (professional and administrative, office, sales and service, laborer and agriculture, unemployed, and others), drinking status (drinker or non-drinker), smoking status (every-day smoker, sometime smoker, ex-smoker, or non-smoker) and mood. Model 4 was adjusted for hypertension, diabetes, hyperlipidemia, coronary heart disease, stroke, chronic digestive disorders (gastric ulcer, duodenal ulcer, superficial gastritis, atrophic gastritis, gastric polyps, hepatitis, cirrhosis, and reflux), and family history of cancers (each yes or no) in addition to the variables included in model 3. Model 5 was adjusted for *H. pylori* infection in addition to the variables included in model 4. The linear trend test was performed by including the median value of each tertile category of the dietary pattern scores as a continuous variable in the model. Tests of statistical significance were two sided and *p* < 0.05 was considered to be statistically significant for all tests.

## Results

### Characteristics of the study population

A total of 2,468 participants were included in this study. Table 1 gives the distribution of 696 cases of gastric cancer and 1,772 controls according to demography, lifestyle and other characteristics. There were differences between cases and controls. Compared with the control subjects, cases were more likely to be younger, male, laborer, smokers, non-drinker and to be given a diagnosis of *H. pylori*-positive, gastric ulcer, duodenal ulcer, superficial gastritis, atrophic gastritis, gastric polyp, reflux, and also were slightly more likely to have a family history of cancers. Additionally, controls were more pleasure, leaner than cases. Furthermore, the case subjects had a significantly higher proportion of individuals with a lower educational status, whereas the proportion of coronary heart disease, stroke, hepatitis, and cirrhosis was similar in both groups (results were shown in Table 1).

**Table 1:** Characteristics of the participants.

	**Total (*****n*** = **2,468)**		***P***
	**Control** **(*****n*** = **1,772)**	**Case** **(*****n*** = **696)**	
**Demography**			
Age (years)	65.39 ± 9.64	63.26 ± 10.06	<0.001
Male, *n* (%)	1,178 (66.48)	515 (73.99)	<0.001
**Education**, ***n*** **(%)**			
Middle school or less	1,495 (84.37)	622 (89.37)	0.003
High school	239 (13.49)	66 (9.48)	
College or more	38 (2.14)	8 (1.15)	
**Occupation**			
Professional, administrative	143 (8.07)	16 (2.30)	<0.001
Office, sales, and service	59 (3.33)	7 (1.01)	
Laborer, agriculture	1,033 (58.30)	414 (59.48)	
Unemployed, others	537 (30.30)	259 (37.21)	
**Lifestyle**			
**Smoking status**, ***n*** **(%)**			<**0.001**
Everyday	473 (26.69)	204 (29.31)	
Sometimes	70 (3.95)	22 (3.16)	
Ex-smoker	185 (10.44)	173 (24.86)	
Non-smoker	1,044 (58.92)	297 (42.67)	
**Drinking status**, ***n*** **(%)**			<0.001
Drinker	819 (46.22)	172 (24.71)	
Non-drinker	953 (53.78)	524 (75.29)	
**Mood**, ***n*** **(%)**			<0.001
Pleasure	1,063 (59.99)	357 (51.29)	
Moderate	623 (35.16)	273 (39.22)	
Unpleasure	86 (4.85)	66 (9.48)	
***H. pylori*** **infection**, ***n*** **(%)**			<0.001
Positive	628 (35.44)	255 (36.64)	
Negative	821 (46.33)	438 (62.93)	
Missing	323 (18.23)	3 (0.43)	
BMI (kg/m^2^)	21.63 ± 3.43	24.97 ± 3.34	<0.001
**Disease**, ***n*** **(%)**			
Hypertension	645 (36.40)	181 (26.01)	<0.001
Diabetes	205 (11.57)	54 (7.76)	0.005
Hyperlipidemia	223 (12.58)	63 (9.05)	0.014
Coronary heart disease	178 (10.05)	65 (9.34)	0.596
Stroke	223 (12.58)	69 (9.91)	0.065
**Chronic digestive disease**			
Gastric ulcer	110 (6.21)	189 (27.16)	<0.001
Duodenal ulcer	38 (2.14)	35 (5.03)	<0.001
Superficial gastritis	255 (14.39)	133 (19.11)	0.004
Atrophic gastritis	27 (1.52)	51 (7.33)	<0.001
Gastric polyp	22 (1.24)	27 (3.88)	<0.001
Hepatitis	38 (2.14)	24 (3.45)	0.062
Cirrhosis	8 (0.45)	3 (0.42)	0.261
Reflux	65 (3.67)	81 (11.64)	<0.001
**Family history of cancers**, ***n*** **(%)**			
Yes	303 (17.10)	170 (24.43)	<0.001

### Major dietary patterns

Table 2 shows factor loadings of the principal component analysis-extracted dietary patterns. The first pattern was characterized by a high intake of flavors, eggs, garlic, vegetables and red meat and therefore was named the flavor, garlic, and protein pattern. The second pattern had high factor loadings for barbecue food, fried food, red meat and seafood and was named the fast-food pattern. The third pattern consisted of high consumption of fruits and vegetables and was named the vegetable and fruit pattern. The fourth pattern was characterized by a high intake of pickled food, processed meat products and soy products and therefore was named the pickled food, processed meat products and soy products pattern. The fifth pattern had high factor loadings for snacks and carbonated drinks and was named the non-staple food pattern. The last pattern consisted of high consumption of coffee and dairy products and was named the coffee and dairy pattern.

**Table 2:** Dietary pattern and factor loading.

**Food group**	**Factor loading**					
	**Flavor, garlic, and protein pattern**	**Fast-food pattern**	**Vegetable and fruit pattern**	**Pickled food, processed meat products, and soy products pattern**	**Non-staple food pattern**	**Coffee and dairy pattern**
Meat	**0.429**	**0.537**	0.154	0.128	0.143	0.089
Seafood	−0.092	**0.480**	0.369	0.324	0.113	0.055
Pickled food	−0.025	0.084	−0.020	**0.696**	0.104	−0.066
Processed meat products	0.069	−0.029	0.004	**0.677**	0.039	0.032
Barbecue food	−0.041	**0.834**	−0.049	−0.029	0.028	0.005
Fried food	0.168	**0.699**	0.017	0.016	−0.044	−0.001
Eggs	**0.611**	0.088	0.042	−0.014	−0.123	0.076
Soy products	0.338	0.122	0.144	**0.450**	−0.209	0.293
Dairy products	0.125	0.138	0.028	−0.163	0.165	**0.616**
Vegetables	**0.455**	0.056	**0.808**	0.015	0.061	0.006
Fruits	−0.064	0.030	**0.929**	−0.025	−0.008	0.022
Garlic	**0.461**	0.058	−0.059	0.299	0.124	0.041
Snacks	0.292	−0.028	0.030	−0.006	**0.722**	−0.029
Flavor	**0.785**	0.028	0.122	−0.031	0.168	−0.065
Carbonated drinks	−0.131	0.092	0.024	0.136	**0.720**	0.043
Coffee	−0.056	−0.083	−0.003	0.092	−0.063	**0.791**

Only foods with factor loading >0.4 are highlighted in bold.

### Association between dietary patterns and risk of gastric cancer

Table 3 described the *OR*s and 95% *CI*s according to the factor score tertiles for each pattern. Five models were used in this analysis. In all five models, the fast-food pattern was associated with an increased risk of gastric cancer [*OR*s (95% *Ci*s) for the highest vs. lowest tertiles: 1.548 (1.040–2.304), 1.927 (1.241–2.991), 2.042 (1.297–3.215), 2.349 (1.456–3.791), 2.133 (1.299–3.502), *p* for trend < 0.01]. The non-staple food pattern was associated with an increased risk of gastric cancer in model 1, model 2, and model 3 [*OR*s (95% *Ci*s) for the highest vs. lowest tertiles: 1.478 (1.106–1.975), 1.449 (1.055–1.991), 1.419 (1.023–1.968), *p* for trend < 0.01]. However, this association was eliminated in model 4 and model 5 [*OR*s (95% *Ci*s) for the highest vs. lowest tertiles: 1.352 (0.957–1.910), 1.149 (0.804–1.642), *p* for trend < 0.01]. The pickled food, processed meat products and soy products pattern was associated with decreased risk of gastric cancer in model 1, model 2, model 3, and model 4 [*OR*s (95% *Ci*s) for the highest vs. lowest tertiles: 0.608 (0.467–0.792), 0.652 (0.484–0.877), 0.666 (0.490–0.906), 0.685 (0.496–0.946), *p* for trend were 0.065 and others all < 0.001, respectively]. However, this association was eliminated in model 5 [*OR*s (95% *CI*s) for the highest vs. lowest tertiles: 0.919 (0.659–1.282), *p* for trend < 0.01]. The coffee and dairy pattern was associated with decreased risk of gastric cancer in model 2, model 4 and model 5 [*OR*s (95% *CI*s) for the highest vs. lowest tertiles: 0.720 (0.522–0.922), 0.660 (0.465–0.937), 0.690 (0.481–0.989), *p* for trend < 0.001]. However, this association was eliminated in model 1 and model 3 [*OR*s (95% *CI*s) for the highest vs. lowest tertiles: 0.850 (0.638–1.134), 0.746 (0.536–1.040), *p* for trend were 0.058 and <0.01, respectively]. On the other hand, no significant association was observed between other patterns and gastric cancer risk.

**Table 3:** Association between dietary patterns and risk for gastric cancer.

	**Model 1**	**Model 2**	**Model 3**	**Model 4**	**Model 5**
**Flavors, garlic, and protein pattern**					
Low	Re	Re	Re	Re	Re
Moderate	1.220 (0.914, 1.628)	1.034 (0.749, 1.426)	1.057 (0.759, 1.473)	0.990 (0.697, 1.405)	1.022 (0.716, 1.460)
High	1.104 (0.750, 1.625)	0.866 (0.563, 1.330)	0.855 (0.548, 1.333)	0.744 (0.467, 1.185)	0.786 (0.488, 1.265)
*P* for trend	0.004	<0.001	<0.001	<0.001	<0.001
**Fast food pattern**					
Low	Re	Re	Re	Re	Re
Moderate	1.243 (0.922,1.674)	1.269 (0.914, 1.762)	1.321 (0.942, 1.853)	1.486 (1.039, 2.124)	1.451 (1.002, 2.101)
High	1.548 (1.040, 2.304)	1.927 (1.241, 2.991)	2.042 (1.297, 3.215)	2.349 (1.456, 3.791)	2.133 (1.299, 3.502)
*P* for trend	0.001	<0.001	<0.001	<0.001	<0.001
**Vegetable and fruit pattern**					
Low	Re	Re	Re	Re	Re
Moderate	0.895 (0.685, 1.170)	1.048 (0.777, 1.414)	1.073 (0.787, 1.462)	1.046 (0.754, 1.450)	1.130 (0.811, 1.575)
High	0.992 (0.697, 1.410)	1.197 (0.808, 1.774)	1.188 (0.791, 1.786)	1.076 (0.703, 1.646)	1.050 (0.682, 1.617)
*P* for trend	0.004	<0.001	<0.001	<0.001	<0.001
**Pickled food, processed meat products and soy products pattern**					
Low	Re	Re	Re	Re	Re
Moderate	0.855 (0.678, 1.078)	0.894 (0.689, 1.159)	0.932 (0.712, 1.220)	0.903 (0.680, 1.201)	1.025 (0.764, 1.376)
High	0.608 (0.467, 0.792)	0.652 (0.484, 0.877)	0.666 (0.490, 0.906)	0.685 (0.496, 0.946)	0.919 (0.659, 1.282)
*P* for trend	0.065	<0.001	<0.001	<0.001	<0.001
**Non-staple food pattern**					
Low	Re	Re	Re	Re	Re
Moderate	1.155 (0.901, 1.479)	1.118 (0.850, 1.470)	1.102 (0.832, 1.459)	1.050 (0.780, 1.414)	0.975 (0.717, 1.327)
High	1.478 (1.106, 1.975)	1.449 (1.055, 1.991)	1.419 (1.023, 1.968)	1.352 (0.957, 1.910)	1.149 (0.804, 1.642)
*P* for trend	<0.001	<0.001	<0.001	<0.001	<0.001
**Coffee and dairy pattern**					
Low	Re	Re	Re	Re	Re
Moderate	0.940 (0.731, 1.207)	0.854 (0.647, 1.127)	0.877 (0.659, 1.168)	0.835 (0.618, 1.130)	0.872 (0.638, 1.193)
High	0.850 (0.638, 1.134)	0.720 (0.522, 0.922)	0.746 (0.536, 1.040)	0.660 (0.465, 0.937)	0.690 (0.481, 0.989)
*P* for trend	0.058	<0.001	<0.001	<0.001	<0.001

All values are OR and 95% CI.

Model 1: Crude model.

Model 2: Adjusted for age, gender and BMI.

Model 3: Additionally adjusted for education, occupation, drinking status, smoking status and mood.

Model 4: Additionally adjusted for hypertension, diabetes, hyperlipidemia, coronary heart disease, stroke, chronic digestive disorders (gastric ulcer, duodenal ulcer, superficial gastritis, atrophic gastritis, gastric polyps, hepatitis, cirrhosis, and reflux), and family history.

Model 5: Additionally adjusted for *H. pylori* infection.

## Discussion

In this case-control study, it was found that (1) the fast-food pattern and the non-staple food pattern may be associated with an increased risk of gastric cancer; (2) the pickled food, processed meat products and soy products pattern and the coffee and dairy products pattern may be associated with decreased risk of gastric cancer; (3) no significant association was observed between other patterns and risk of gastric cancer; (4) associations between dietary patterns and gastric cancer existed the linear tendency. The above findings could be summarized as a higher intake of specific foods might be associated with the risk of gastric cancer.

Our research has shown that the fast-food pattern and the non-staple food pattern could increase the risk of gastric cancer. The first had a higher consumption of barbecue food, fried food, red meat and seafood. The latter had a higher consumption of snacks and carbonated drinks. The results of ours were consistent with many studies (14, 18–20). There were several mechanisms to explain this association: (1) when red meat was cooked at high temperatures, genotoxic N-nitroso compounds, heterocyclic amines and polycyclic aromatic hydrocarbons could be produced, which were well-known carcinogens (10, 14, 19, 20). (2) Heme iron, which was contained in red meat might play a role in gastric carcinogenesis through the endogenous formation of carcinogenic N-nitroso compounds, DNA damage and oxidative stress (20, 21). Furthermore, iron was a critical factor for bacterial growth in *H. pylori*, which was a well-known risk factor for gastric cancer (20, 21). (3) Snacks usually contained saturated fats, which could induce the expression of certain inflammatory mediators associated with carcinogenesis (14). (4) Participants who consumed more carbonated drinks tended to have reflux symptoms (22). (5) Carbonated drink usually contained more sugar, which could lead to obesity (7). Obesity was conducive to chronic low-grade inflammation, hyperinsulinemia, hyperleptinemia, and an elevated production of endogenous sex steroid hormones, all of which might contribute to tumor growth (7).

Our research has shown that the pickled food, processed meat products and soy products pattern and the coffee and dairy pattern could decrease the risk of gastric cancer. The first had a high intake of pickled food, processed meat products and soy products. The latter had a high intake of coffee and dairy products. Different from other studies, our research has shown that a higher consumption of pickled food, processed meat products and soy products could decrease the risk of gastric cancer (20, 23). Pickled food and processed meat products usually were recognized as foods that could increase the risk of gastric cancer (20, 23). One side was attributed to salt that was added in pickled food and the other side was attributed to formation of carcinogens (23, 24). The different results may be explained by this important factor. Although pickled food and processed meat products had the largest factor loading, soy products also accounted for a large proportion. The actual intake amount of soy products was higher than that of pickled food and processed meat products. Many studies found that a higher intake of soy products could decrease the risk of gastric cancer (25, 26). Therefore, the promoting effect of pickled food and processed meat products on the risk of gastric cancer may be covered by the protective effect of soy products on the risk of gastric cancer in this dietary pattern. The benefits of soybean intake could be due to two factors. First, soybean and soy products contained isoflavones, which had anti-inflammatory and anti-oxidative effects (26, 27). Furthermore, some experimental studies suggested that genistein, a kind of isoflavones, could induce apoptosis of human gastric carcinoma cells and inhibit *H. pylori* growth (26). Second, soybean and soy products also included saponins, which were reported as an antitumor ingredient (27). Similar to other studies, our research has shown that a higher consumption of coffee and dairy products could decrease the risk of gastric cancer (28, 29). There were several mechanisms to explain this association: (1) coffee was a mix of bioactive compounds. It contained phenolic compounds and two lipids: cafestol and kahweol. These compounds could suppress cancer growth through antioxidant, anti-genotoxic activity, mitochondrial toxicity and anti-inflammatory environmental regulation (28). (2) Dairy products contained several components, including vitamin D, minerals, calcium and conjugated linoleic acid. The protective effects of these components on gastric cancer may be due to antitumor effects (30, 31). (3) Fermented dairy products such as cheese and yogurt contain lactic acid bacteria, which could suppress the growth of *H. pylori* by producing inhibitory substances, including lactic acid and bacteriocin (30, 31).

## Strengthen and limitations

This study has some strengths. First, we used dietary patterns in our study. Compared to a single food, the dietary pattern could provide a more comprehensive assessment of the collective importance of different dietary factors. Second, all potential confounding factors were adjusted in our study. Last, this was a case-control study based on community. There also existed several potential limitations in this study. First, although our questionnaire requested detailed information regarding the consumption of food and food groups, it was a short version that included only 16 food groups. Second, the actual intake of various foods could not be estimated through the questionnaire, and recall bias was inevitable (32). Recalled diet was found to be more closely associated with past diet than with current diet, recall over many years contained errors due to failures in memory. Third, prevalence cases were included in this study. The prevalence-incidence bias was inevitable. Fourth, the data on total energy intake were not available in this study and it was not adjusted as a confounder. Fifth, confounding would distort the strength of the association (33). Although some potential confounders in this analysis were adjusted for, it was likely that some residual confounding remained due to imperfect statistical adjustment, imperfect confounder measurement, and the existence of confounders that were unknown, unmeasured or otherwise unadjusted for (33). Sixth, there existed the missing value of *H. pylori* in our study, which was a great contributor to increase the risk of gastric cancer. Seventh, participants were from the Huaihe River Basin, the applicability was limited. Last, due to the limitation of the observational study, causation could not be detected in this study.

## Conclusions

In conclusion, it was indicated that specific dietary patterns may be associated with the risk of gastric cancer. This study had considerable public health implications. The quality of diet was important to health and longevity (34). Actually, the dietary pattern was associated with health behaviors, lifestyle and sociodemographic factors, it reflected individual awareness and attitudes toward health (34, 35). Participants who preferred an unhealthy diet need to receive dietary education and diet management for the prevention of gastric cancer. Societies and individuals might success in lowering their risk for gastric cancer by changing preferences in diet. Additional prospective studies with more participants were needed.

## Data availability statement

The original contributions presented in the study are included in the article/supplementary material, further inquiries can be directed to the corresponding authors.

## Ethics statement

The study complied with the principles of the Declaration of Helsinki and was approved by the Ethics Committee of National Center for Chronic and Noncommunicable Disease Control and Prevention, Chinese Center for Disease Control and Prevention. In addition, all participants were aware of the research purpose and provided written informed consent. The patients/participants provided their written informed consent to participate in this study.

## Author contributions

XW: data analysis, writing—original draft, and writing—review and editing. QZ, WZ, and WW: writing—review and editing. HG: writing—original draft and writing—review and editing. NW: conceptualization, investigation, project administration, and supervision. XF and BZ: investigation and supervision. ZF: conceptualization and writing—review and editing. JW: conceptualization, methodology, project administration, supervision, and writing—review and editing. All authors contributed to the article and approved the submitted version.
